# Complete genome sequence of *Anaerococcus prevotii* type strain (PC1^T^)

**DOI:** 10.4056/sigs.24194

**Published:** 2009-09-24

**Authors:** Kurt LaButti, Rudiger Pukall, Katja Steenblock, Tijana Glavina Del Rio, Hope Tice, Alex Copeland, Jan-Fang Cheng, Susan Lucas, Feng Chen, Matt Nolan, David Bruce, Lynne Goodwin, Sam Pitluck, Natalia Ivanova, Konstantinos Mavromatis, Galina Ovchinnikova, Amrita Pati, Amy Chen, Krishna Palaniappan, Miriam Land, Loren Hauser, Yun-Juan Chang, Cynthia D. Jeffries, Patrick Chain, Elizabeth Saunders, Thomas Brettin, John C. Detter, Cliff Han, Markus Göker, Jim Bristow, Jonathan A. Eisen, Victor Markowitz, Philip Hugenholtz, Nikos C Kyrpides, Hans-Peter Klenk, Alla Lapidus

**Affiliations:** 1 DOE Joint Genome Institute, Walnut Creek, California, USA; 2 DSMZ - German Collection of Microorganisms and Cell Cultures GmbH, Braunschweig, Germany; 3 Los Alamos National Laboratory, Bioscience Division, Los Alamos, New Mexico, USA; 4 Biological Data Management and Technology Center, Lawrence Berkeley National Laboratory, Berkeley, California, USA; 5 Oak Ridge National Laboratory, Oak Ridge, Tennessee, USA; 6 Lawrence Livermore National Laboratory, Livermore, California, USA; 7 University of California Davis Genome Center, Davis, California, USA

**Keywords:** *Firmicutes*, *Clostridiales*, ‘*Peptostreptococcaceae’*, Gram-positive, coccoid, human oral microflora, skin, non-motile, non-sporulating, anaerobic

## Abstract

*Anaerococcus prevotii* (Foubert and Douglas 1948) Ezaki *et al.* 2001 is the type species of the genus, and is of phylogenetic interest because of its arguable assignment to the provisionally arranged family ‘*Peptostreptococcaceae*’. *A. prevotii* is an obligate anaerobic coccus, usually arranged in clumps or tetrads. The strain, whose genome is described here, was originally isolated from human plasma; other strains of the species were also isolated from clinical specimen. Here we describe the features of this organism, together with the complete genome sequence and annotation. This is the first completed genome sequence of a member of the genus. Next to *Finegoldia magna, A. prevotii* is only the second species from the family *‘Peptostreptococcaceae’* for which a complete genome sequence is described. The 1,998,633 bp long genome (chromosome and one plasmid) with its 1852 protein-coding and 61 RNA genes is a part of the *** G****enomic* *** E****ncyclopedia of* *** B****acteria and* *** A****rchaea * project.

## Introduction

*Anaerococcus prevotii* strain PC1^T^ (= DSM 20548 = ATCC 9321 = JCM 6508) is the type strain of the species and the type species of the genus [1]. Six strains of the species were characterized by Foubert and Douglas in 1948, originally designated as ‘*Micrococcus prevotii*’, but subsequently placed in the genus *Peptococcus* [2]. Based on a comparative study published by Ezaki *et al*. [3], the type strain of *P. prevotii* was then transferred to the genus ‘*Peptostreptococcus’* and later on assigned to the novel genus *Anaerococcus* as *A. prevotii* [1]. The organism is a Gram-positive, anaerobic, indole-negative coccus. The major metabolic end product from metabolism of peptone-yeast-glucose (PYG) is butyric acid. *A. prevotii* was provisionally assigned to the arranged family ‘*Peptostreptococcaceae*’ within the order *Clostridiales*, also designated as Family XI *Incertae sedis* [4]. Here we present a summary classification and a set of features for *A. prevotii* strain PC1^T^ together with the description of the complete genomic sequencing and annotation.

## Classification and features

Within the last few years, several changes occurred in the classification of the anaerobic Gram-positive cocci. There are currently five genera of anaerobic Gram-positive cocci which may be isolated from humans (*Peptostreptococcus*, *Peptoniphilus, Parvimonas*, *Finegoldia*, and *Anaerococcus*). Members of the species *A. prevotii* are frequently recovered from human clinical specimens such as vaginal discharges and ovarian, peritoneal, sacral or lung abscesses. In particular, *A. prevotii* was also described as a common isolate of the normal flora of skin, the oral cavity and the gut [3]. Historically the Gram-positive anaerobic cocci were identified mainly by using phenotypic traits, but as shown by Song *et al.*, this often led to the misidentification of *A. vaginalis* strains, which were mistakenly assigned to *A. prevotii* or *A. tetradius* [5]. Currently Genbank does not contain any16S rRNA sequences from cultivated strains that can be clearly linked to the species *A. prevotii* with over 95% gene sequence similarity. Recently, the temporal diversity of the human skin microbiome was analyzed using 16S rRNA gene phylotyping. It is noteworthy that several clones originated from different skin sites (gluteal crease, occiput, umbilicus, popliteal fossa, volar forearm). These isolates were taken from two patients and showed close relationships to *A. prevotii* [6]. No closely related isolates or uncultivated clones with more than 84% 16S rRNA gene sequence identity are recorded from global ocean screenings and environmental samples (except for human skin).

**Figure 1:**
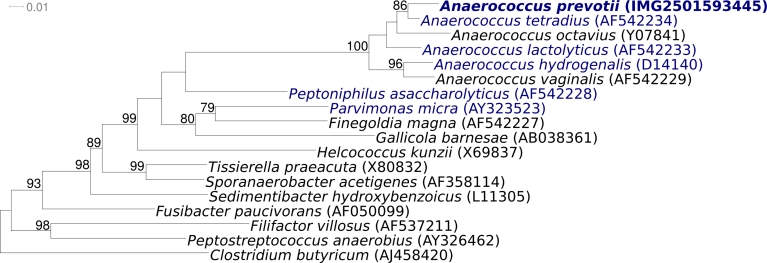
Phylogenetic tree highlighting the position of *A. prevotii* PC1^T^ relative to all type strains of the genus *Anaerococcus* and the type strains of all other genera within the family ‘*Peptostreptococcaceae’* inferred from 1,302 aligned characters [7,8] of the 16S rRNA sequence under the maximum likelihood criterion [9]. Rooting was done with the type species of the genus *Clostridium*. The branches are scaled in terms of the expected number of substitutions per site. Numbers above branches are support values from 1,000 bootstrap replicates if larger than 60%. Lineages with type strain genome sequencing projects registered in GOLD [10] are shown in blue, published genomes in bold.

Figure 1 shows the phylogenetic neighborhood of *A. prevotii* strain PC1^T^ in a 16S rRNA based tree. The four 16S rRNA gene copies in the genome of strain PC1^T^ differ by up to 15 nucleotides from each other, and by up to 9 nucleotides from the previously published 16S rRNA sequence generated from strain CCUG 41932 (AF542232). The difference between the genome data and the reported 16S rRNA gene sequence is most likely due to sequencing errors in the previously reported sequence data.

**Figure 2:**
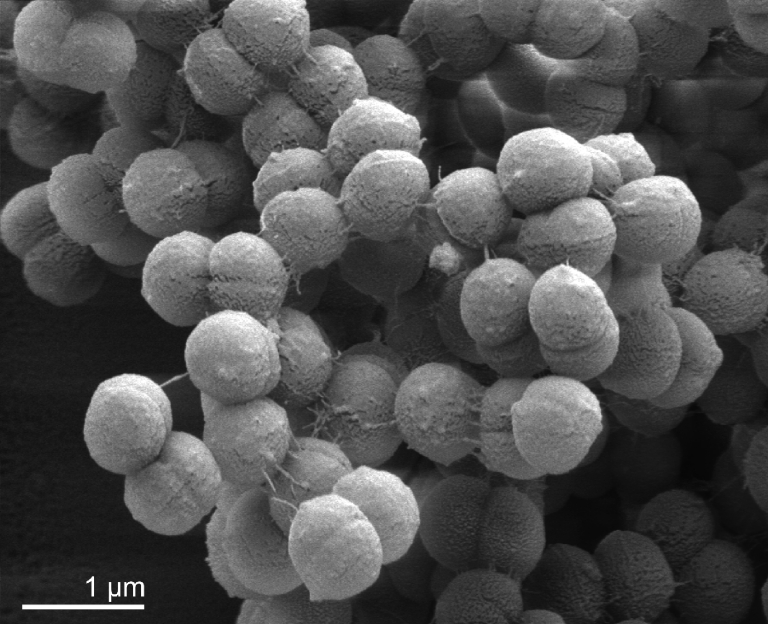
Scanning electron micrograph of *A. prevotii* PC1^T^ (M. Rohde, HZI Braunschweig)

*A. prevotii* PC1^T^ cells are Gram-positive and non-motile(Table 1). Cells grown in PYG broth are 0.6-0.9 µm in diameter and occur in pairs, tetrads or irregular clumps or short chains (Figure 2). Colonies range from 0.5 to 2 mm in diameter on Columbia blood agar. Optimum temperature for growth is 37°C. Strain PC1^T^ metabolizes peptones and amino acids and the major metabolic end product from PYG medium is butyric acid. Most species of the genus *Anaerococcus* ferment carbohydrates weakly. *A. prevotii* is proteolytic. α-Glucosidase, α –galactosidase, ß-glucuronidase and pyroglutamyl arylamidase activities are detectable [19,20]. Production of urease may vary among strains of the species. Most strains produce ammonia from threonine and serine [3] by deamination of the amino acids to pyruvate. *A. prevotii* is resistant to sodium polyanethol sulfonate [21], but susceptible to the penicillins [19].

**Table 1:** Classification and general features of *A. prevotii* PC1^T^ in accordance with the MIGS recommendations [11]

MIGS ID	Property	Term	Evidence code
	Current classification	Domain *Bacteria*	TAS [12]
		Phylum *Firmicutes*	TAS [13]
		Class *Clostridia*	TAS [4]
		Order *Clostridiales*	TAS [14]
		Family *‘Peptostreptococcaceae’*	TAS [4]
		Genus *Anaerococcus*	TAS [1]
		Species *Anaerococcus prevotii*	TAS [1]
		Type strain PC1	TAS [1,3]
	Gram stain	positive	TAS [15]
	Cell shape	coccoid	TAS [15]
	Motility	nonmotile	TAS [15]
	Sporulation	nonsporulating	TAS [15]
	Temperature range	mesophile	TAS [15]
	Optimum temperature	37°C	TAS [15]
	Salinity	growth in PYG +6% NaCl	TAS [16]
MIGS-22	Oxygen requirement	anaerobic	TAS [15]
	Carbon source	unknown	
	Energy source	peptones	TAS [1,3]
MIGS-6	Habitat	human mouth, skin and vaginal microflora	TAS [3,10]
MIGS-15	Biotic relationship	free living	NAS
MIGS-14	Pathogenicity	opportunistic infections	TAS [10]
	Biosafety level	2	TAS [17]
	Isolation	human plasma	TAS [3]
MIGS-4	Geographic location	not reported	
MIGS-5	Sample collection time	not reported	
MIGS-4.1 MIGS-4.2	Latitude – Longitude	not reported	
MIGS-4.3	Depth	not reported	
MIGS-4.4	Altitude	not reported	

Evidence codes - IDA: Inferred from Direct Assay (first time in publication); TAS: Traceable Author Statement (i.e., a direct report exists in the literature); NAS: Non-traceable Author Statement (i.e., not directly observed for the living, isolated sample, but based on a generally accepted property for the species, or anecdotal evidence). These evidence codes are available from the Gene Ontology project [18]. If the evidence code is IDA, then the property was directly observed for a live isolate by one of the authors or an expert mentioned in the acknowledgements.

### Chemotaxonomy

Cell wall amino acid analysis of strain PC1^T^ yielded peptidoglycan type A4α′, composed of L-Lys-D-Glu [22], type A12.2 according to the DSMZ catalogue of strains. Cell wall sugars are glucose, glucosamine and galactose [22]. Major cellular fatty acid composition of the type strain was analyzed by Lambert and Armfield in 1979 [23] and by Ezaki et al. in 1983 [3], but the results of these studies are contradictory. No other chemotaxonomic data are available at present.

## Genome sequencing and annotation

### Genome project history

This organism was selected for sequencing on the basis of its phylogenetic position, and is part of the *** G****enomic* *** E****ncyclopedia of* *** B****acteria and* *** A****rchaea * project. The genome project is deposited in the Genomes OnLine Database [10] and the complete genome sequence has been deposited in GenBank. Sequence, finishing and annotation were performed by the DOE Joint Genome Institute (JGI). A summary of the project information is shown in Table 2.

**Table 2:** Genome sequencing project information

MIGS ID	Property	Term
MIGS-31	Finishing quality	Finished
MIGS-28	Libraries used	Three genomic libraries: two Sanger libraries - 8 kb pMCL200 and fosmid pcc1Fos - and one 454 pyrosequence standard library
MIGS-29	Sequencing platforms	ABI3730, 454 GS FLX, Illumina GA
MIGS-31.2	Sequencing coverage	6.8 Sanger; 42.3 pyrosequence
MIGS-30	Assemblers	Newbler version 1.1.02.15, Arachne
MIGS-32	Gene calling method	Prodigal, GenePRIMP
	Genbank IDs	CP001708 (chromosome)
		CP001709 (plasmid pAPRE01)
	Genbank Dates of Release	4/28/2009 (chromosome)
		4/28/2009 (plasmid pAPRE01)
	GOLD ID	Gc01089
	NCBI project ID	29533
	Database: IMG-GEBA	2501533213
MIGS-13	Source identifier	DSM 20548
	Project relevance	Tree of Life, GEBA

### Growth conditions and DNA isolation

*A. prevotii* strain PC1^T^, DSM 20548, was grown anaerobically in DSMZ medium 104 [24] at 37°C. DNA was isolated from 1-1.5 g of cell paste using Qiagen Genomic 500 DNA Kit (Qiagen, Hilden, Germany) following the instructions given by the manufacturer, but with a modified protocol for cell lysis, LALMP, according to Wu *et al.* [25].

### Genome sequencing and assembly

The genome was sequenced using a combination of Sanger, 454 and Illumina sequencing platforms. All general aspects of library construction and sequencing can be found at the JGI web site. Reads produced by 454 Pyrosequencing were assembled using the Newbler assembler version 1.1.02.15 (Roche). Large Newbler contigs were broken into 2,196 overlapping fragments of 1,000 bp and entered into the assembly as pseudo-reads. The sequences were assigned quality scores based on Newbler consensus q-scores with modifications to account for overlap redundancy and to adjust inflated q-scores. A hybrid 454/Sanger assembly was made using the Arachne assembler. Possible mis-assemblies were corrected and gaps between contigs were closed by custom primer walks from sub-clones or PCR products. A total of 66 Sanger finishing reads were produced. Illumina reads were used to improve the final consensus quality using an in-house developed tool (the Polisher). The final assembly consisted of 18,576 Sanger and 464,157 Roche/454 reads. The error rate of the completed genome sequence is less than 1 in 100,000. Together all sequence types provided 49.1 coverage of the genome.

### Genome annotation

Genes were identified using Prodigal [26] as part of the Oak Ridge National Laboratory genome annotation pipeline, followed by a round of manual curation using the JGI GenePRIMP pipeline [27]. The predicted CDSs were translated and used to search the National Center for Biotechnology Information (NCBI) nonredundant database, UniProt, TIGRFam, Pfam, PRIAM, KEGG, COG, and InterPro databases. Additional gene prediction analysis and functional annotation was performed within the Integrated Microbial Genomes (IMG-ER) platform [28].

## Genome properties

**Figure 3:**
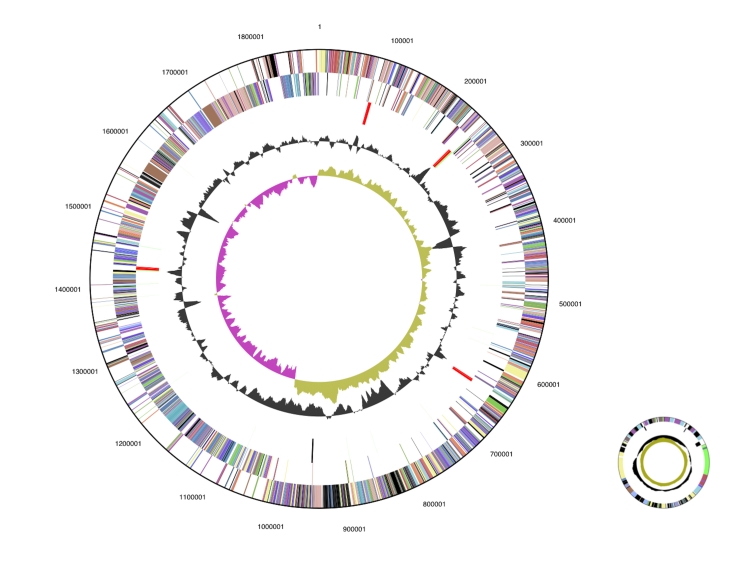
Graphical circular map of the genome. From outside to the center: Genes on forward strand (color by COG categories), Genes on reverse strand (color by COG categories), RNA genes (tRNAs green, sRNAs red, other RNAs black), GC content, GC skew.

The genome is 1,998,633 bp long (chromosome and one circular plasmid) with a 35.6% GC content (Table 3). Of the 1,913 genes predicted, 1,852 were protein coding genes, and 61 were RNAs. A total of 46 pseudogenes were also identified, with 73.1% of the genes being assigned a putative function. The remaining genes were annotated as hypothetical proteins. The distribution of genes into COGs functional categories is presented in Figure 3 and Table 4.

**Table 3:** Genome Statistics

**Attribute**	**Value**	**% of Total**
Genome size (bp)	1,998,633	100.00%
DNA Coding region (bp)	1,815,671	90.85%
DNA G+C content (bp)	712,291	35.64%
Number of replicons	2	
Extrachromosomal elements	1	
Total genes	1913	100.00%
RNA genes	61	3.19%
rRNA operons	4	
Protein-coding genes	1852	96.81%
Pseudo genes	46	2.405%
Genes with function prediction	1399	73.13%
Genes in paralog clusters	231	12.08%
Genes assigned to COGs	1421	74.28%
Genes assigned Pfam domains	1428	74.65%
Genes with signal peptides	337	17.62%
Genes with transmembrane helices	467	24.41%
CRISPR repeats	0	

**Table 4:** Number of genes associated with the 21 general COG functional categories

**Code**	**Value**	** %age**	**Description**
J	133	7.2	Translation, ribosomal structure and biogenesis
A	0	0.0	RNA processing and modification
K	118	6.4	Transcription
L	105	5.7	Replication, recombination and repair
B	1	0.1	Chromatin structure and dynamics
D	20	1.1	Cell cycle control, mitosis and meiosis
Y	0	0.0	Nuclear structure
V	55	3.0	Defense mechanisms
T	43	2.3	Signal transduction mechanisms
M	69	3.7	Cell wall/membrane biogenesis
N	5	0.3	Cell motility
Z	0	0.0	Cytoskeleton
W	0	0.0	Extracellular structures
U	19	1.0	Intracellular trafficking and secretion
O	61	3.3	Posttranslational modification, protein turnover, chaperones
C	84	4.5	Energy production and conversion
G	144	7.8	Carbohydrate transport and metabolism
E	107	5.8	Amino acid transport and metabolism
F	61	3.3	Nucleotide transport and metabolism
H	56	3.0	Coenzyme transport and metabolism
I	37	2.0	Lipid transport and metabolism
P	102	5.5	Inorganic ion transport and metabolism
Q	9	0.5	Secondary metabolites biosynthesis, transport and catabolism
R	84	4.5	General function prediction only
S	118	6.4	Function unknown
-	431	23.3	Not in COGs
